# Brazilian version of the Brief Screener for Substance and Behavioral Addiction

**DOI:** 10.3389/fpsyg.2025.1642665

**Published:** 2025-08-22

**Authors:** Cristiane Faiad, João Marôco, Hermano Tavares, David Hodgins, Lucas Heiki Matsunaga

**Affiliations:** ^1^The Institute of Psychology, The University of Brasília, Brasília, Brazil; ^2^Intrepid Lab & CETRAD, ECEO, Universidade Lusófona, Lisboa, Portugal; ^3^Department of Psychiatry, Universidade de Sao Paulo, São Paulo, Brazil; ^4^Department of Psychology, University of Calgary, Calgary, AB, Canada; ^5^Tokyo College, The University of Tokyo, Tokyo, Japan

**Keywords:** instrument adaptation, dependent behaviors, addictions, excessive behaviors, evidence of validity

## Abstract

**Introduction:**

Excessive and compulsive behaviors, including substance and behavioral addictions, represent a growing global concern. In Brazil, the increasing prevalence of these behaviors underscores the need for effective screening tools to identify individuals at risk. The Brief Screener for Substance and Behavioral Addiction (SSBA) has been recognized internationally for its utility in both clinical assessment and public health surveillance. This study aimed to adapt the SSBA for use in Brazil, with potential applications in other Portuguese-speaking countries.

**Methods:**

The adaptation process followed international guidelines for cross-cultural adaptation of psychometric instruments. It included forward translation into Portuguese, back-translation into English, and expert committee review to ensure semantic and conceptual equivalence. A pilot study was conducted to assess clarity and relevance. Subsequently, the Brazilian version of the SSBA was administered to a sample of 450 individuals, comprising both clinical and non-clinical populations. Psychometric analyses evaluated the instrument’s reliability, validity, and factorial structure.

**Results:**

The Brazilian version of the SSBA demonstrated good internal consistency and satisfactory construct validity across subscales. Confirmatory factor analysis supported the original structure of the instrument, and no major linguistic or cultural adaptations were required. The screener showed strong discriminative power between clinical and non-clinical participants, indicating its effectiveness for identifying individuals at risk for addiction-related disorders.

**Discussion:**

The adapted SSBA is a reliable and valid tool for the Brazilian context and may be extended to other Lusophone countries. It provides a brief yet comprehensive screening method suitable for various settings, including clinical practice, research, and community health. The instrument is particularly valuable for health professionals working in addiction prevention, diagnosis, and treatment, supporting early identification and intervention efforts.

## Brazilian version of the Brief Screener for Substance and Behavioral Addiction

One major challenge for researchers and health professionals is understanding extraordinary behaviors (EB), whether addictive or behavioral. These behaviors are typically excessive, repetitive, and driven by impulses that are hard to resist. They often serve as a form of escapism or relief from stress and anxiety, yet persist despite causing harm to an individual’s life and relationships (Demetrovics et al., 2022; Grant et al., 2013; Karim and Chaudhri, 2012). Understanding EB is crucial for developing effective interventions and clearly defining their boundaries and impact.

To guide research and clinical interventions, Brand et al. (2022) caution against over-pathologizing normal behaviors based solely on frequency and propose three criteria for identifying potentially addictive behaviors. First, the behavior must have clinical relevance, causing harm or dysfunction. Second, it should align theoretically with addiction. Third, there must be empirical evidence, gathered through tools like self-reports, interviews, and experiments, supporting its biological and psychological basis. Despite their caution, the authors stress the need for greater investment in treatment and public health initiatives.

A systematic review by Mudry et al. (2011) highlighted the lack of consensus around excessive behavior syndrome, which includes addictions like internet use, sex, compulsive eating, substance abuse, gaming, overworking, shopping, trichotillomania, and extreme exercise. The nosological classification of many of these behaviors remains unclear, as they are not explicitly recognized in the DSM-5 or ICD-11, though some fall under broader diagnostic categories (Demetrovics et al., 2022; Starcevic and Khazaal, 2017). Research also shows that certain individuals, particularly those with depression, anxiety, or ADHD, are more prone to these behaviors (Demetrovics et al., 2022).

Further, numerous studies have explored different types of excessive or addictive behaviors, such as gaming (Burleigh et al., 2019; Kim et al., 2022), shopping (Guerrero-Vaca et al., 2019), sex (Brand et al., 2016), pornography and internet use (Monteiro et al., 2020; Souza and Cappellozza, 2019), eating (Santos, 2023), and alcohol use (Hill and Mazurek, 2003; Romera et al., 2022). Despite this progress, a key challenge—especially in Brazil—remains the development of reliable tools to measure and assess these behaviors.

The SSBA is increasingly used in research to assess addiction risk across a range of behaviors and substances. Developed by Schluter et al. (2020), it was based on symptom reports from individuals with lived experience of addiction and initially covered ten behaviors, including substance use, gambling, eating, shopping, sex, gaming, and work. Hodgins et al. (2023) later expanded it to 13 behaviors by three more categories: opioid use, excessive exercise, and compulsive working. In a study with 656 college students, they confirmed the SSBA’s validity and reliability, highlighting its value as a tool for both research and clinical assessment.

Given the lack of screening tools for behavioral addictions in Brazil, this study adapts the SSBA to the Brazilian context, establishes content validity through expert evaluation, and examines its initial factor structure in a community sample. Moreover, to effectively adapt and validate a tool like the SSBA for use in Brazil, we essentially consider the cultural, social, and behavioral norms that shape how excessive behaviors are manifested and perceived. This is especially important because the Brazilian society presents unique contexts, such as widespread internet use alongside limited access to mental health services (Andrade et al., 2020), high levels of religiosity that influence moral attitudes toward behaviors like sex and substance use (Moreira-Almeida et al., 2014), and pronounced socioeconomic inequality that may increase vulnerability to stress-related coping behaviors (Patel et al., 2018). These factors can influence both the prevalence and reporting of excessive behaviors, introducing potential biases in measurement. Cross-cultural validity is therefore critical in adapting psychological instruments for Brazil, requiring more than linguistic translation to ensure conceptual and functional equivalence (Borsa et al., 2012). Without these adaptations, assessment tools may fail to capture the lived experience of behavioral addiction in diverse Brazilian populations, undermining their utility for research and public health policy.

Further, to address the complexity and cultural relevance of excessive behaviors in Brazil, this study extended the original SSBA by including eight additional behaviors that are commonly reported in clinical or social contexts as problematic or potentially addictive. These addictions (e.g., excessive dedication to a romantic partner, cosmetic procedures, and use of social media) were informed by qualitative insights from Brazilian clinicians and researchers, as well as national trends in behavior-related complaints and treatment-seeking patterns. The inclusion of these behaviors is consistent with the criteria proposed by Brand et al. (2022), as they have been linked in the literature to functional impairment (e.g., Andreassen et al., 2012), impulsivity and loss of control (e.g., Hormes et al., 2014), and neurobiological correlates similar to those found in substance-related addictions (e.g., Turel et al., 2014). Moreover, these behaviors align with a growing body of international literature advocating for a culturally sensitive and empirically grounded expansion of the behavioral addiction framework (Müller et al., 2019). Therefore, the adaptation process did not merely translate the scale but also aimed to reflect behaviors with plausible addictive characteristics that are particularly salient in the Brazilian context.

Thus, the goal of this study is to lay the groundwork for broader applications of the SSBA beyond Western, industrialized populations. By addressing gaps in assessment tools, it aims to enhance clinical practices, support preventive measures, and encourage adoption and cultural adaptation of the SSBA in other Lusophone countries (e.g., Angola, Cape Verde, Mozambique, Portugal, Timor-Leste). Especially, this research is timeless due to the globally growing mental health concerns associated with addiction.

## Materials and methods

The Brief Screener for Behavioral Addiction (SSBA), developed by Schluter et al. (2018), assesses the risk of substance and behavioral dependence in adults over reflection on the past 12 months. It focuses on behaviors that lead to significant problems, using four core statements for each behavior or substance: (a) “*I did it too much*,” (b) “*Once I started, I couldn’t stop*,” (c) “*I felt I needed to do this to function*,” and (d) “*I continued despite the problems it caused.*” Responses are rated on a 6-point Likert scale (0 = Never to 5 = I didn’t do any of these), with options to skip or decline to answer. To strengthen selection validity, Schluter et al. (2020) and Hodgins et al. (2023) recommend adding questions on psychological treatment, medication use, and hospitalization.

The original version included ten variables: alcohol, marijuana, tobacco, psychostimulants, gambling, overeating, shopping, sex, video games (Schluter et al., 2018). A second version by Hodgins et al. (2023) expanded to more behaviors: opioids, physical exercise, and study. Additionally, a new version of the Screener for Substance and Behavioral Addictions (SSBA-G) was developed by Thege et al. (2023). For the version adapted for Brazil, Schluter et al.’s (2018) original version was used. However, after a critical analysis of behaviors by the research team, eight additional factors were subsequently incorporated. These include excessive work, self-harm, excessive devotion to a romantic partner, outbursts of anger, hair-pulling or body hair removal, skin picking, theft, excessive internet use, and the use of tranquilizers. In this study, these extraordinary behaviors will be referred to as behavioral addictions (BAs). The specific characteristics of the 21 substances and behaviors are outlined in Table 1.

**TABLE 1:** Behavioral addictions.

Behavioral addictions	Description
(1) Alcohol	Beer, wine, etc.
(2) Marijuana	Medicinal and non-medicinal use of marijuana, hashish, THC/hash oil, etc.
(3) Tobacco	Cigarettes, cigars, and other tobacco/nicotine products (e.g., vaporizers, electronics).
(4) Psychostimulants	Crack cocaine, powder cocaine (blow/snow/snort), speed, diet pills, ecstasy, methamphetamine (solid/crystallized), etc., as well as the medicinal and non-medical use of prescription amphetamine-type stimulants (e.g., Ritalin).
(5) Gambling	Slot machine games, online gambling, casino games, lotteries, and any other bets for cash/other rewards.
(6) Overeating	Consumption of food in quantities that exceed what is necessary for daily sustenance and good health.
(7) Shopping	Any in-store or online purchase.
(8) Sexual activity	Sexual activity (e.g., intercourse, oral sex, masturbation), as well as excessive and/or inappropriate use of online or offline (e.g., pornography, phone sex).
(9) Video games	Single/multiplayer video games, played online or offline on all types of platforms and consoles (e.g., PlayStation, PC), handhelds (e.g., Switch), as well as mobiles and computers.
(10) Opioids	It involves heroin, fentanyl, etc., as well as prescription painkillers such as morphine, methadone, codeine, OxyContin, Vicodin, etc.
(11) Excessive	Any form of physical exercise that exceeds what is necessary to maintain good health and meet physical demands.
(12) Over-studying	All study activities beyond regular academic obligations and assignments from coursework.
(13) Overwork	Any paid or voluntary work beyond formal duties or capacity, such as during a weekend/day off/workday or in the evening/at home, etc.
(14) Self-harm	Cutting, hitting, or burning behaviors.
(15) Dedication to romantic partner	Involves excessive dedication to the partner
(16) Outbursts of anger	Involves behaviors such as shouting, swearing, breaking objects, or physical aggression.
(17) Pulling out hair and/or body hair.	Performed with hands, tweezers, or other instruments.
(18) Picking own skin	Actions performed with hands, tweezers, or other instruments.
(19) Theft/kleptomania	It involves stealing items that do not belong to you, whether from stores, workplaces, or the homes of acquaintances.
(20) Internet use	It involves using social media, watching videos, following news. Does not include pornography, gambling, online video games or online shopping. Access to social media (e.g., Instagram, Whatsapp, Youtube, TikTok), for long periods of time.
(21) Tranquilizers	Sleeping pills such as Rivotril, Valium, Lexotan, Stilnox, Ptaz, Dormonid or black band medications obtained with(out) a blue prescription.

### Psychopathological and sociodemographic assessment

To evaluate validity evidence, the Reduced Taxonomy of Psychopathology Screening Scale (ER-HiTOP-R) was applied. This instrument is a shortened version of the 57-item ER-HiTOP (Oliveira and Corrêa, in press), based on the Hierarchical Taxonomy of Psychopathology (HiTOP) model (Krueger et al., 2018). The HiTOP model proposes a dimensional, hierarchical classification of mental disorders grounded in empirical evidence. The ER-HiTOP-R consists of 31 items rated on a 5-point scale (1 = “Never,” 5 = “Always”) and assesses 11 dimensions. Negative Affects (NA) measures frequent and intense experiences of sadness, anxiety, irritability, emotional instability, and suicidal thoughts (5 items, α = 0.85, ω = 0.89). Fear Disorders (MD) assesses fear responses to various situations, people, and objects (5 items, α = 0.81, ω = 0.83). Eating pathology (EA) evaluates disordered eating behaviors, including restriction, binge-eating, and purging (5 items, α = 0.78, ω = 0.84). Sexual disorders (SD) identifies sexual dysfunctions related to arousal, desire, orgasm, pain, and aversion (6 items, α = 0.87, ω = 0.91). Somatic complaints (QS) measures the frequency and intensity of physical discomforts such as headaches, gastrointestinal issues, and neurofunctional problems (5 items, α = 0.78, ω = 0.82). Interpersonal distancing (ID) assesses social withdrawal, discomfort in social settings, and lack of social initiative or pleasure in interpersonal interactions (5 items, α = 0.80, ω = 0.82). Thought disorders (TD) identifies perceptual disturbances, including hallucinations, delusions, and eccentric thoughts or behaviors (5 items, α = 0.64, ω = 0.69). Manic symptoms (MS) captures elevated mood, increased energy, impulsiveness, aggression, and cognitive hyperactivity (5 items, α = 0.72, ω = 0.79). Antisocial behavior (AB) characterizes rule-breaking, dishonesty, rebellion, and aggressive tendencies (5 items, α = 0.76, ω = 0.78). Antagonistic externalizing (AE) assesses abusive relationships through manipulative behaviors, egocentrism, grandiosity, insensitivity, and attention-seeking (5 items, α = 0.80, ω = 0.83). Disinhibited externalizing (DE) identifies impulsive, reckless behaviors, including substance abuse, irresponsibility, and risk-taking (6 items, α = 0.74, ω = 0.77).

A sociodemographic questionnaire was also administered to collect data on age, sex, gender, education, and clinical history. The clinical history section included questions about the use of controlled medications, current participation in therapy, and any history of psychiatric hospitalization.

### SSBA adaptation process for Brazil

The initial version of the SSBA was translated into Brazilian Portuguese by a researcher, psychiatrist, and professor at a Brazilian university. Then, the instrument was independently translated by two bilingual psychologists and subsequently reviewed by a psychology research group. These versions were then synthesized into a third version, which was back-translated into English. The adaptation process was supervised by one of the scale’s original authors, Hidden for Anonymous Review, ensuring accuracy and reviewing both existing items and those added specifically for the Brazilian version.

### Participants and data collection procedure

Participants were recruited through announcements on social media by convenience. The survey was accessed via a link on the Microsoft platform. The average response time was 22 min. This survey was reviewed and approved by the Research Ethics Committee Hidden for Anonymous Review, and all participants provided informed consent before completing the instruments. A total of 415 adults answered the survey, with a mean age of 43.33 years (SD = 13.43), and 323 (77.8%) were women, 92 (22.2%) men, and two identified as non-binary. Of the 415 adult participants, 270 (65.1%) identified as white, 15 (3.6%) as black, and 110 (26.5%) as mixed races. Regarding marital status, 118 (28.4%) were single, 188 (45.3%) were married, 60 (14.5%) were in a stable union, and 35 (8.4%) were divorced. The majority worked (309, 74.5%) in different areas of activity, distributed across different regions of Brazil. Regarding clinical profile, 133 (32%) were undergoing psychological treatment at the time of the survey, 77 (18.6%) were undergoing psychiatric treatment, and 87 (21%) were taking psychiatric medication. Of these, 87 (21%) had some mental health diagnosis, and 9 (2.2%) had been admitted to a psychiatric clinic for mental health reasons (see Table 2).

**TABLE 2:** Descriptive data of research participants (*N* = 450).

Variable	*n*	%	Variable	*N*	%	Variable	*n*	%
**Sex**			**Occupation**			Undergraduate student	43	10.4
Woman	321	77.3	Retired	34	8.2	Teacher	35	8.4
Man	92	22.2	Unemployed	6	1.4	Private company	65	15.7
Non-binary	2	0.5	Stay-at-home	6	1.4	Others	93	22.4
**Color/race/ethnicity**			**Study/internship**	48	11.6	Public safety	46	11.1
White	270	65.1	Sick leave	2	0.5	Public service	131	31.6
Black	15	3.6	Other	10	2.4	**Internet usage**		
Brown	110	26.5	Work	309	74.5	Up 2 h/day	173	41.7
Yellow	9	2.2	**Family income**			Between 3 and 5 h/day	168	40.5
Indigenous	6	1.4	Up to R$ 1,839.95	14	3.4	Between 6 and 8 h/day	52	12.5
Other	2	0.5	Up to R$ 10,450.96	92	22.2	More than 8 h/day	21	5.1
I prefer not to declare	3	0.7	Up to R$ 22,435.43	123	29.6			
**Marital status**			**Payment**					
Single	118	28.4	Up to R$ 3,086.88	15	3.6			
Stable union	60	14.5	Up to R$ 5,524.29	46	11.1			
Married	188	45.3	Up to R$ 847.69	5	1.2			
Separated	6	1.4	More than R$ 22,435.43	120	28.9			
Divorced	35	8.4						
Widower	7	1.7						
I prefer not to declare	1	0.2						

### Data analysis

Data analysis was conducted to evaluate the psychometric properties of the adapted SSBA, addressing the study’s aims of assessing content validity, internal structure, and associations with external variables. As a preliminary step, the distribution of item responses was examined to characterize the data. Descriptive statistics, including mean, standard deviation, quartiles, skewness, and kurtosis, were used to evaluate the items’ distributional properties. To gather evidence of content validity, a panel of experts conducted a judge-based evaluation of the items, followed by the application of the instrument to the target sample. A content validity index (CVI) of 80% or higher was considered acceptable.

Evidence of internal structure was assessed using confirmatory factor analysis (CFA) with the weighted least squares mean and variance adjusted (WLSMV) estimator for ordinal items. Factor loadings equal to or greater than 0.30 were considered acceptable indicators of item-factor relationships. CFA was conducted using the *lavaan* package (Rosseel, 2012), while descriptive analyses were performed using the *skmr* package (Waring et al., 2025). Reliability was evaluated using Cronbach’s alpha and McDonald’s omega, computed with the *semTools* package (Jorgensen and Johnson, 2022).

Model fit was assessed using standard goodness-of-fit indices: root mean square error of approximation (RMSEA, with reference values < 0.10), Comparative Fit Index (CFI > 0.90), and Tucker-Lewis Index (TLI > 0.90), following recommendations by Hair et al. (2009). Finally, validity evidence based on relationships with external variables was established through correlations between SSBA scores and the HiTOP psychopathology measure.

## Results

### Validity evidence based on the internal structure of the SSBA

In the analysis of the distribution properties of the items, the data in Table 3 indicate a violation of univariate normality in the items of substance use. Only shopping, internet use, and excessive commerce behaviors had scores between 3 and 4. The results suggest that, for most items, the behaviors or addictions were not prevalent among the respondents, except for psychostimulant use.

**TABLE 3:** Distribution properties.

Item	M	SD	Min	P_25_	Mdn	P_75_	Max	sk	ku	Hist
Alcohol 1	1.586	0.956	0	1	1	2.0	5	0.624	0.118	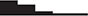
Alcohol 2	1.092	0.713	0	1	1	1.0	5	2.298	8.338	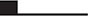
Alcohol 3	1.292	0.890	0	1	1	1.0	5	1.595	3.358	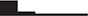
Alcohol 4	1.108	0.739	0	1	1	1.0	5	2.083	6.722	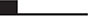
Tobacco 1	1.012	0.830	0	1	1	1.0	5	2.282	8.022	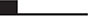
Tobacco 2	0.966	0.819	0	1	1	1.0	5	2.643	10.112	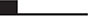
Tobacco 3	0.961	0.795	0	1	1	1.0	5	2.720	11.429	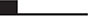
Tobacco 4	1.019	0.940	0	1	1	1.0	5	2.677	8.868	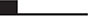
Psychost 1	0.814	0.483	0	1	1	1.0	4	0.330	5.744	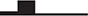
Psychost 2	0.805	0.443	0	1	1	1.0	3	−0.676	1.437	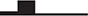
Psychost 3	0.841	0.559	0	1	1	1.0	5	1.783	12.623	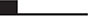
Psychost 4	0.829	0.522	0	1	1	1.0	5	1.439	13.182	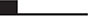
Gambling 1	0.949	0.677	0	1	1	1.0	5	1.922	8.794	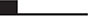
Gambling 2	0.867	0.564	0	1	1	1.0	5	2.234	16.359	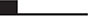
Gambling 3	0.867	0.555	0	1	1	1.0	5	2.233	17.095	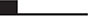
Gambling 4	0.855	0.528	0	1	1	1.0	5	1.824	15.064	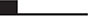
Shopping 1	1.942	0.993	0	1	2	3.0	5	0.484	0.036	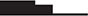
Shopping 2	1.296	0.832	0	1	1	1.0	5	1.939	5.045	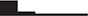
Shopping 3	1.508	0.955	0	1	1	2.0	5	1.295	1.999	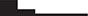
Shopping 4	1.325	0.913	0	1	1	1.0	5	2.033	4.873	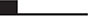
Videoga 1	1.347	1.050	0	1	1	1.5	5	1.551	2.533	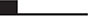
Videoga 2	1.229	0.987	0	1	1	1.0	5	2.013	4.878	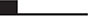
Vídeoga 3	1.181	0.952	0	1	1	1.0	5	2.079	5.346	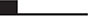
Vídeoga 4	1.128	0.868	0	1	1	1.0	5	2.202	6.738	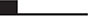
Eating 1	2.181	1.060	0	1	2	3.0	5	0.207	−0.183	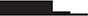
Eating 2	1.800	1.132	0	1	1	2.0	5	0.985	0.478	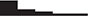
Eating 3	1.795	1.137	0	1	1	2.5	5	0.956	0.432	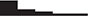
Eating 4	1.788	1.195	0	1	1	2.5	5	1.039	0.423	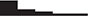
Sex 1	1.458	1.013	0	1	1	2.0	5	1.359	1.640	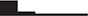
Sex 2	1.296	0.954	0	1	1	1.0	5	1.848	3.707	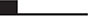
Sex 3	1.439	1.043	0	1	1	2.0	5	1.476	1.941	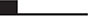
Sex 4	1.205	0.900	0	1	1	1.0	5	2.163	5.431	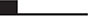
Work 1	2.193	1.232	0	1	2	3.0	5	0.311	−0.837	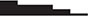
Work 2	1.752	1.144	0	1	1	3.0	5	0.929	−0.039	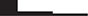
Work 3	1.720	1.229	0	1	1	2.0	5	1.126	0.445	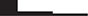
Work 4	1.723	1.237	0	1	1	2.0	5	1.147	0.388	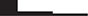
Self-mut 1	0.851	0.587	0	1	1	1.0	5	1.683	10.402	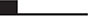
Self-mut 2	0.834	0.576	0	1	1	1.0	5	2.058	14.963	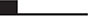
Self-mut 3	0.872	0.663	0	1	1	1.0	5	2.522	14.501	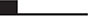
Self-mut 4	0.853	0.645	0	1	1	1.0	5	2.682	16.562	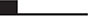
Partner 1	1.708	1.148	0	1	1	2.5	5	0.698	−0.144	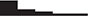
Partner 2	1.325	0.979	0	1	1	1.0	5	1.563	2.636	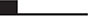
Partner 3	1.523	1.105	0	1	1	2.0	5	1.201	1.085	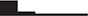
Partner 4	1.318	1.002	0	1	1	1.0	5	1.688	3.012	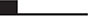
Internet 1	2.858	1.168	0	2	3	4.0	5	−0.077	−0.627	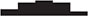
Internet 2	2.402	1.233	0	1	2	3.0	5	0.321	−0.830	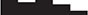
Internet 3	2.024	1.257	0	1	2	3.0	5	0.778	−0.354	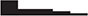
Internet 4	2.111	1.312	0	1	2	3.0	5	0.685	−0.711	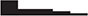
AngerExp 1	1.752	0.975	0	1	2	2.0	5	0.776	0.533	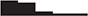
AngerExp 2	1.439	0.909	0	1	1	2.0	5	1.433	2.277	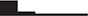
AngerExp 3	1.434	0.953	0	1	1	2.0	5	1.668	3.090	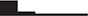
AngerExp 4	1.395	0.908	0	1	1	2.0	5	1.616	2.800	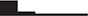
Hair-pulling 1	0.966	0.728	0	1	1	1.0	5	2.186	9.047	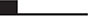
Hair-pulling 2	0.947	0.720	0	1	1	1.0	5	2.554	11.894	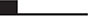
Hair-pulling 3	0.930	0.693	0	1	1	1.0	5	2.696	14.144	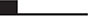
Hair-pulling 4	0.918	0.687	0	1	1	1.0	5	2.874	15.518	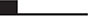
Skin picking 1	1.373	1.060	0	1	1	2.0	5	1.328	1.600	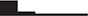
Skin picking 2	1.333	1.097	0	1	1	1.0	5	1.519	1.917	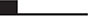
Skin picking 3	1.212	0.970	0	1	1	1.0	5	1.835	3.902	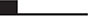
Skin picking 4	1.275	1.043	0	1	1	1.0	5	1.727	3.013	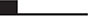
Exercise 1	1.422	0.949	0	1	1	2.0	5	1.491	2.818	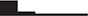
Exercise 2	1.248	0.850	0	1	1	1.0	5	2.091	5.710	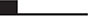
Exercise 3	1.516	1.116	0	1	1	2.0	5	1.438	1.578	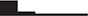
Exercise 4	1.135	0.699	0	1	1	1.0	5	2.431	9.592	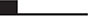
Study 1	1.636	1.026	0	1	1	2.0	5	0.956	0.531	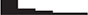
Study 2	1.354	0.899	0	1	1	1.5	5	1.691	3.159	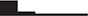
Study 3	1.523	1.107	0	1	1	2.0	5	1.508	1.881	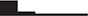
Study 4	1.255	0.858	0	1	1	1.0	5	2.209	5.914	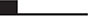

The histogram provides a visual representation of the response distribution for each item.

The dimensionality of the SSBA was assessed using CFA to evaluate the model’s fit to the data. The latent variable behavioral addictions was represented by 19 observed variables, as listed in Table 1, with opium and marijuana removed due to high collinearity with psychostimulants.

Model adjustments were based on the polychoric correlation matrix of the items, using the WLSMV estimation method with oblique rotations. The analysis was conducted in Rusing the “lavaan” package. The measurement model demonstrated a satisfactory fit to the data {χ^2^ = 5,527.793, *p* < 0.001; χ^2^/df = 2.665; *n* = 450; CFI = 0.888; NFI = 0.834; TLI = 0.877; SRMR = 0.004; RMSEA = 0.063; *p*(RMSEA ≤ 0.05) < 0.001; 90% CI [0.061, 0.065]}. Factor loadings ranged from 0.694 to 0.980, as shown in Table 4. Scale reliability indicated satisfactory internal consistency, with estimates ranging from 0.839 to 0.975.

**TABLE 4:** Factor loadings.

Item	Load	Factor	α	ω	Item	Load	Factor	α	ω
Alcohol 1	0.694	Alcohol	0.840	0.839	Self-mut 1	0.956	Self-mut	0.973	0.974
Alcohol 2	0.781				Self-mut 2	0.954			
Alcohol 3	0.704				Self-mut 3	0.918			
Alcohol 4	0.880				Self-mut 4	0.980			
Tobacco 1	0.944	Tobacco	0.962	0.964	Partner 1	0.846	Partner	0.937	0.937
Tobacco 2	0.940				Partner 2	0.913			
Tobacco 3	0.904				Partner 3	0.904			
Tobacco 4	0.939				Partner 4	0.900			
Psychost 1	0.966	Psychost	0.943	0.932	Internet 1	0.812	Internet	0.910	0.912
Psychost 2	0.977				Internet 2	0.900			
Psychost 3	0.823				Internet 3	0.818			
Psychost 4	0.786				Internet 4	0.862			
Gambling 1	0.851	Gambling	0.945	0.945	AngerExp 1	0.786	Anger Exp	0.908	0.909
Gambling 2	0.940				AngerExp 2	0.905			
Gambling 3	0.878				AngerExp 3	0.809			
Gambling 4	0.957				AngerExp 4	0.887			
Shopping 1	0.709	Shopping	0.881	0.883	Hair-pulling 1	0.938	Hair Pulling	0.975	0.974
Shopping 2	0.873				Hair-pulling 2	0.950			
Shopping 3	0.815				Hair-pulling 3	0.953			
Shopping 4	0.855				Hair-pulling 4	0.967			
Vídeog 1	0.871	Videog	0.936	0.938	Skin picking 1	0.918	Skin picking	0.967	0.968
Vídeog 2	0.949				Skin picking 2	0.959			
Vídeog 3	0.838				Skin picking 3	0.925			
Vídeog 4	0.902				Skin picking 4	0.951			
Eating 1	0.831	Eating	0.932	0.934	Exercise 1	0.898	Exercise	0.904	0.914
Eating 2	0.920				Exercise 2	0.908			
Eating 3	0.865				Exercise 3	0.821			
Eating 4	0.906				Exercise 4	0.781			
Sex 1	0.839	Sex	0.932	0.933	Study 1	0.830	Study	0.908	0.911
Sex 2	0.930				Study 2	0.905			
Sex 3	0.882				Study 3	0.833			
Sex 4	0.878				Study 4	0.838			
Work 1	0.844	Work	0.918	0.917					
Work 2	0.931								
Work 3	0.815								
Work 4	0.845								

### HiTOP model validation through statistical correlations

**FIGURE 1:**
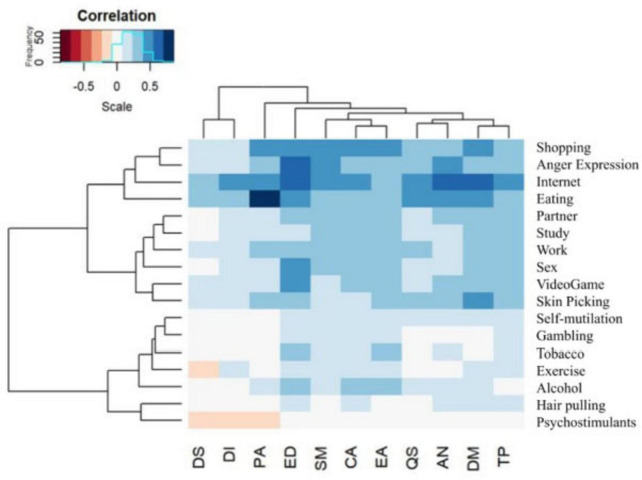
Correlations between the instruments.

In the proposal for the clinical utility of the HiTOP model (Ruggero et al., 2019), convergence between the BSSA and the HiTOP-R was proven, through the *lavInspect* and *gplots* packages. The data indicated serious and positive correlations between AN and Shopping (*rr* = 0.358); excessive Internet use (*rr* = 0.676) and Anger expression (*rr* = 0.455); between DM and Shopping (*r* = 0.436); Eating (*r* = 0.444); Work (*r* = 0.341), Internet (*r* = 0.608); Anger expression (*r* = 0.385); Skin picking (*r* = 0.401) and Study (*r* = 0.329); between PA and Shopping (*r* = 0.445); Overeating (*r* = 0.857); Work (*r* = 0.311); Internet (*r* = 0.476); Anger Expression (*r* = 0.361); between ID and Internet (*r* = 0.413); between SQ and Eating (*r* = 0.457); Work (*r* = 0.372); Internet (*r* = 0.449) and Anger expression (*r* = 0.323); between TP and Shopping (*r* = 0.307); Video game (*r* = 0.333); Eating (*r* = 0.348); Sex (*r* = 0.334); Work (*r* = 0.323); Partner (*r* = 0.344); Internet (*r* = 0.508); Anger expression (*r* = 0.384); Skin picking (*r* = 0.359) and Study (*r* = 0.339); ED and Alcohol (*r* = 0.327); Shopping (*r* = 0.537); Video Game (*r* = 0.408); Eating (*r* = 0.532); Sex (*r* = 0.439); Internet (*r* = 0.683); Anger expression (*r* = 0.595) and Skin picking (*r* = 0.311); between CA and Alcohol (*r* = 0.353); Shopping (*r* = 0.398); Video game (*r* = 0.373); Eating (*r* = 0.336); Work (*r* = 0.304); Partner (*r* = 0.315); Internet (*r* = 0.448); Anger expression (*r* = 0.364); SM and Shopping (*r* = 0.409); Eating (*r* = 0.328); Work (*r* = 0.319); Partner (*r* = 0.333); Internet (*r* = 0.474); Anger outburst (*r* = 0.404); Study (*r* = 0.318) and EE and Alcohol (*r* = 0.323); Tobacco (*r* = 0.330); Shopping (*r* = 0.431); Video games (*r* = 0.384); Eating (*r* = 0.300); Sex (*r* = 0.348); Internet (*r* = 0.389); Expression of anger (*r* = 0.369) and Skin picking (*r* = 0.304). There was no significant manifestation in Behavioral Dependence and Substance Addictions on Psychostimulants, Gambling, Self-mutilation, Hair pulling and Exercise. Figure 1 represents the strongest correlations between the instruments.

## Discussion

Among the challenges of developing more effective interventions for behavioral addictions and substance use in Brazil, the adaptation of the SSBA and the initial studies on its validity represent significant contributions. These efforts enhance the field and provide professionals with better tools for assessment and intervention. The SSBA studies indicate that the diagnostic categories allow for the identification of reliable measures with strong psychometric indicators. In addition to external evidence supporting the scale’s internal consistency, studies on content validity during the adaptation process and its convergence with psychopathology measures further reinforce the instrument’s reliability.

The SSBA structure remained consistent, though psychostimulant, opium, and cannabinoid use measures exhibited high collinearity. Future studies with clinical samples may reveal structural refinements, aligning with the findings of Hodgins et al. (2023). Regarding internal consistency, the SSBA demonstrated excellent reliability indicators, with alpha values exceeding 0.90, corroborating the findings of Thege et al. (2023).

Behavioral addictions generally demonstrate strong psychometric indices and align with psychopathological aspects, making the instrument a promising, brief, and effective screening tool. It can assess the functional impact of addictions, including both emotional and behavioral aspects, as highlighted by Thege et al. (2023), supporting future diagnoses and health interventions.

Despite these contributions, some limitations must be acknowledged. The use of convenience sampling may have introduced selection bias, reducing the generalizability of results beyond the studied population. Additionally, the absence of clinical samples limits conclusions about the SSBA’s sensitivity and specificity in detecting clinically significant cases. Furthermore, gender imbalance in the sample, specifically the underrepresentation of men, warrants attention, as it may obscure potential gender-specific patterns. Future research should consider stratified or male-focused sampling to enhance representativeness. In addition, we acknowledge the role of self-selection bias, as individuals with greater interest or concern about the topic may have been more likely to participate. These limitations suggest caution in interpreting the findings and underscore the importance of future validation studies with diverse and clinical populations to improve the instrument’s diagnostic precision and applicability.

Nevertheless, the SSBA’s potential for clinical and policy use is notable. As a brief, psychometrically sound screening tool, it can support early detection strategies in primary care, mental health, and educational settings. Its ability to capture both behavioral and emotional dimensions of addiction aligns well with integrative care models and public health frameworks. For policymakers, the availability of a validated measure tailored to the Brazilian context fills an important gap, enabling more accurate population-level monitoring and informing the development of targeted prevention and intervention programs.

## Final remarks

The measurement of addictive behaviors in Brazil is limited, with no valid screening studies to date. The SSBA, supported by validity evidence, can be a valuable tool in clinical and health settings. Future research in Portuguese-speaking countries should apply the scale in diverse contexts, such as police forces, schools, and universities, to support public policy and management strategies. A key limitation of this study is its use of a convenience sample, which does not reflect clinical populations. Future research should address this by including samples that represent specific addictive behaviors, like substance use. To establish norms, future studies should broaden sampling in Brazil to include clinical and non-clinical groups and invest in creating normative tables and ROC curve analyses, as recommended by Schluter et al. (2020).

## Data Availability

The data analyzed in this study is subject to the following licenses/restrictions: Request by email. Requests to access these datasets should be directed to crisfaiad@gmail.com.
